# Comparisons of Objective Sleep Quality Between Elderly Individuals With and Without Cataract Surgery: A Cross-Sectional Study of the HEIJO-KYO Cohort

**DOI:** 10.2188/jea.JE20140201

**Published:** 2015-08-05

**Authors:** Kenji Obayashi, Keigo Saeki, Kimie Miyata, Tomo Nishi, Nobuhiro Tone, Nahoko Ogata, Norio Kurumatani

**Affiliations:** 1 Department of Community Health and Epidemiology, Nara Medical University School of Medicine, Nara, Japan; 1 奈良県立医科大学 地域健康医学講座; 2 Department of Ophthalmology, Nara Medical University School of Medicine, Nara, Japan; 2 奈良県立医科大学 眼科学講座; 3 Center for Academic Industrial and Governmental Relations, Nara Medical University School of Medicine, Nara, Japan; 3 奈良県立医科大学 産学官連携推進センター

**Keywords:** sleep quality, cataract surgery, circadian rhythms, actigraphy, melatonin

## Abstract

**Background:**

Cataract surgery (CS) drastically increases the capacity for light reception to the retina. Several previous studies have suggested the beneficial effect of CS on subjectively measured sleep quality; however, the association between CS and objectively measured sleep quality remains uncertain.

**Methods:**

To evaluate the association between CS and objectively measured sleep quality in home settings, we conducted a cross-sectional study in 1037 elderly individuals (mean age, 71.9 years). We evaluated actigraphically measured sleep quality, urinary 6-sulfatoxymelatonin excretion, and ambulatory light levels, in addition to CS status.

**Results:**

The CS group (*n* = 174) showed significantly higher sleep efficiency and shorter wake after sleep onset than the no CS group (*n* = 863), even after adjustment for age, gender, body mass index, current smoking status, alcohol consumption, hypertension, diabetes, sleep medication, bedtime, rising time, daytime physical activity, daytime and nighttime light exposure, and urinary 6-sulfatoxymelatonin excretion (sleep efficiency: 85.8% in the CS group vs 84.4% in the no CS group, *P* = 0.042; wake after sleep onset: 45.7 min vs 50.6 min, respectively, *P* = 0.033). In contrast, urinary 6-sulfatoxymelatonin excretion, sleep onset latency, total sleep time, and sleep-mid time did not differ significantly between the CS and no CS groups.

**Conclusions:**

Among a community-dwelling elderly population, CS is significantly associated with objectively measured sleep quality, but urinary levels of melatonin metabolite do not differ between individuals with and without CS. These associations are independent of daily light exposure profiles.

## Abstract (ja)

**背景:**

白内障手術により網膜への光受容は劇的に増加する。先行研究で白内障手術が主観的に測定された睡眠の質を改善することが報告されているが、白内障手術と客観的睡眠の質の関連は十分に分かってない。

**方法:**

白内障手術と客観的に測定された睡眠の質の関連を明らかにするために、我々は高齢者1037人（平均年齢71.9歳）を対象に横断研究を実施した。白内障手術の有無に加えて、アクチグラフによる睡眠の質、夜間尿中メラトニン主要代謝産物排泄量（6-sufatoxymelatonin）、および環境照度を測定した。

**結果:**

白内障手術群（n = 174）は非白内障手術群（n = 863）と比較して、年齢、性別、BMI、喫煙、飲酒、高血圧、糖尿病、睡眠薬の使用、入床時刻、離床時刻、日中身体活動量、日中および夜間の光曝露量を調整した後でも、有意に睡眠効率が高く、睡眠後覚醒時間が短かった（睡眠効率：85.8% vs. 84.4%, P = 0.042; 睡眠後覚醒時間：45.7分 vs. 50.6分, P = 0.033）。対照的に、夜間尿中メラトニン主要代謝産物排泄量、睡眠潜時、総睡眠時間、および睡眠中央時刻は2群で有意な差を認めなかった。

**結論:**

一般高齢者集団において、白内障手術の有無は睡眠効率や睡眠後覚醒時間と有意に関連していた。しかし、夜間尿中メラトニン主要代謝産物排泄量は白内障手術群と非白内障手術群で有意な差を認めなかった。また、これらの関連は日常の光曝露量と独立していた。

## INTRODUCTION

Sleep problems occur frequently in the elderly.^1^ Previous epidemiological studies suggest that approximately 40% of elderly individuals reported sleep problems related to sleep initiation and maintenance.^2^^,^^3^ Poor sleep is associated with increased risk of depression, dementia, cardiovascular diseases, and mortality.^4^^–^^7^ Therefore, the significant public health burden associated with sleep problems in the elderly is increasing and affecting the aging society.

Light is a primary environmental cue for the suprachiasmatic nucleus of the hypothalamus, which is an essential component of the master biological clock.^8^ Using light information, the suprachiasmatic nucleus regulates circadian alignment between internal biological rhythm and environmental rhythm. In a previous experimental study, bright light intervention during daytime (2500 lux for 4 hours) in elderly patients with insomnia significantly increased melatonin secretion, which is a pineal gland hormone related to good sleep quality.^9^^,^^10^ In addition, a recent randomized controlled trial in elderly patients with major depressive disorder revealed that morning bright light intervention with short wavelengths (7500 lux for 1 hour) significantly improved sleep quality measured using actigraphy.^11^

Aging significantly progresses cloudiness in the crystalline lens.^12^ Age-related decreases in pupil area and crystalline lens transmission cause a decrease in light reception to the retina in phakic individuals.^13^ Elderly individuals aged 70 years are estimated to have roughly 20% of the capacity for light reception to the retina compared with that of teens. In addition, human lens aging is particularly associated with a loss of shorter wavelengths (below 500 nm), which are most sensitive for intrinsically photosensitive retinal ganglion cells, a primary light receptor in the retina.^12^ Thus, light reception sensitive to circadian biological rhythms will decrease in elderly individuals without advanced cataract. Cataract surgery (CS), replacement of clouded crystalline lenses with intraocular lenses, drastically increases the capacity for light reception to the retina. As a result, elderly individuals with CS could have a greater capacity for light reception to the retina than those without CS.

Several previous studies reporting the effect of CS on subjective sleep quality suggest that intraocular lens implantation significantly improves sleep quality measured using self-reported questionnaires at 1, 2, 6, and 9 months after CS,^14^^–^^18^ although these studies did not take daily light exposure profiles into account. To the best of our knowledge, there are no previous studies assessing the effect of CS on objective sleep quality, except for a Japanese study with only fifteen cataract patients.^19^ This study reported no significant differences in actigraphically measured sleep quality before and after CS, but this could be a result of low statistical power due to a small sample size.

Thus, CS may increase melatonin secretion and improve objective sleep quality. To explore the associations of CS with melatonin secretion and objective sleep quality, we conducted this cross-sectional study with 1037 community-dwelling elderly individuals where urinary melatonin levels and actigraphic sleep parameters were measured in individuals with and without CS and associations were assessed using statistical models adjusted for daily light exposure profiles.

## METHODS

### Participants

We recruited community-dwelling elderly individuals aged ≥60 years with the cooperation of local residents’ associations and elderly residents’ clubs in Nara, Japan, and a total of 1127 elderly subjects were voluntarily enrolled with a gift card incentive (participation rate unknown) between September 2010 and March 2014 in the Housing Environments and Health Investigation among Japanese Older People in Nara, Kansai Region: a prospective community-based cohort (HEIJO-KYO) study.^20^ In total, 1037 home-dwelling participants met the following inclusion criteria: completion of the CS questionnaire, underwent urinary 6-sulfatoxymelatonin excretion (UME) measurements, and underwent actigraphic measurements. All participants provided written informed consent. The study protocol was performed in accordance with the ethics committee of Nara Medical University.

### Ascertainment of CS

A standardized questionnaire was used to ascertain if participants received CS in at least one eye. Accuracy of self-reported CS in the initial 194 participants (18.7%) was assessed by an ophthalmologist’s direct observation of the IOL using slit lamp examination. Agreement regarding CS history between the two data sets was sufficiently high (Kappa coefficient = 0.95).

### Measurements of objective sleep quality

An actigraph (Actiwatch 2; Respironics Inc., Murrysville, PA, USA), worn on the non-dominant wrist, was used to measure objective sleep quality at 1-minute intervals during two consecutive nights. Sleep/awake status at each epoch, sleep onset, and sleep offset were automatically detected by Actiware version 5.5 (Respironics Inc.) with the default algorithm.^21^ Epochs with higher activity counts than a moderate threshold (40 counts/minute) were treated as awake. Sleep onset was defined as the first minute followed by a 10-minute immobility period comprising not more than one epoch with any motion count. Sleep offset was defined as the last minute following a 10-minute period of immobility.

Objective data (awake/sleep status and sleep onset/offset) and self-reported data (bedtime and rising time) were used to assess the following five actigraphic sleep parameters for each night: (1) total sleep time, the time spent with sleep (below the activity threshold of 40 counts/minute) between sleep onset and sleep offset; (2) sleep efficiency, the percentage calculated from total sleep time divided by the time between bedtime and rising time, derived from self-reported sleep diary; (3) wake after sleep onset, the time spent awake (above the activity threshold of 40 counts/minute) between sleep onset and rising time; (4) sleep onset latency, the time between bedtime and sleep onset; and (5) sleep-mid time, the mid-time between sleep onset and sleep offset.

### UME measurement

The overnight urine collection protocol involved discarding the last void at bedtime and collecting each subsequent void, including the first morning void. Samples were stored in dark bottles with a cold pack at room temperature. Total volume was measured, and samples were stored at −20°C until the assay (within a few months). Urinary 6-sulfatoxymelatonin concentrations were measured using a highly sensitive enzyme-linked immunosorbent assay kit (RE54031; IBL International, Hamburg, Germany). UME was calculated as follows: UME (µg) = 6-sulfatoxymelatonin concentration (µg/mL) × total overnight urine volume (mL). UME was used as an index of melatonin secretion based on evidence that UME correlates closely with secreted levels of this hormone.^22^ The reproducibility of UME among the initial 192 participants was assessed by an additional urine collection approximately 4 months later. The intra-individual coefficient of variation was calculated using mean values of the first and second measurements and their standard deviation (SD). The inter-individual coefficient of variation was calculated using mean values of the two measurements of each participant and their SD. At 4 months, the intra-individual coefficient of variation was 2.1%, the inter-individual coefficient of variation was 29.9%, and the intraclass correlation coefficient between the two UME levels in the 188 participants (four missing) was 0.66 (95% confidence interval, 0.57–0.73).

### Measurement of daily light exposure profiles

Daytime and nighttime light exposure were measured at 1-minute intervals during two consecutive days, using a wrist light meter (Actiwatch 2; Respironics Inc.), worn on the non-dominant wrist, and a portable light meter (LX-28SD; Sato Shouji Inc., Kanagawa, Japan) set in the bedroom, respectively. All participants were instructed not to cover the sensor with their clothing and to roll up their sleeves using special rubber bands. Values <1 lux during the out-of-bed period were considered artifacts due to clothing covering the sensor and were not included in the analyses.^23^ When the duration of missing data exceeded half of the out-of-bed period, parameters were treated as blank data. Daytime light exposure was defined as mean light intensity from rising time to bedtime, and nighttime light exposure was defined as mean light intensity from bedtime to rising time. In our previous study,^21^ day-to-day correlations of daytime and nighttime light exposure between the two days were moderately high (Spearman’s rank correlation coefficient = 0.61 and 0.66, respectively).

### Other measurements

Body mass index (BMI) was calculated as weight (kg) divided by height (m) squared (kg/m^2^). Current smoking status, habitual alcohol consumption, and sleep medication use were evaluated by administering a questionnaire to participants. Hypertension was defined on the basis of medical history and current antihypertensive therapy. Diabetes mellitus was defined on the basis of the following assessments: medical history, current antidiabetic therapy, fasting plasma glucose levels ≥7.0 mmol/L, and glycated hemoglobin levels ≥6.5% according to the National Glycohemoglobin Standardization Program value. Daytime physical activity was calculated as mean of physical activity counts over the two days, evaluated using the actigraph from rising time to bedtime.

### Statistical analyses

Variables with a normal distribution were expressed as the mean (SD), whereas asymmetrically distributed variables were reported as the median (interquartile range). Means and medians were compared between the CS and no CS groups using the unpaired *t*-test and Mann-Whitney U test, respectively. The chi-square test was used for comparing categorical data. Variables, including age, gender, BMI, current smoking status, alcohol consumption, hypertension, diabetes, sleep medication, bedtime, rising time, daytime physical activity, daytime and nighttime light exposure, and UME, were compared between the CS and no CS groups. Independent variables were mutually adjusted in multivariate models, using analysis of covariance, where there was no serious multicollinearity (all variance inflation factors <10). Mean values of bedtime, rising time, daytime physical activity, daytime and nighttime light exposure, and actigraphic sleep parameters on two consecutive days were used for analyses. Daytime light exposure, UME, and sleep onset latency with a skewed distribution were naturally log-transformed for analyses. Statistical analyses were performed using SPSS version 19.0 for Windows (IBM SPSS Inc., Chicago, IL, USA). A two-tailed *P* value <0.05 was considered significant.

## RESULTS

The mean (SD) age of participants was 71.9 (7.1) years, and 481 individuals (46.4%) were male. Of 1037 participants, 174 participants (16.8%) had received CS. Participants were divided into two age groups (≥75 and <75 years). The CS group showed significantly higher age than the no CS group in both age groups (Table 1). Among the older population (≥75 years), daytime light exposure was significantly lower in the CS group than in the no CS group. UME did not significantly differ between the CS and no CS groups. Also, mean nighttime bedroom temperature did not significantly differ between the two groups (13.0°C in the CS group vs 12.8°C in the no CS group; *P* = 0.65).

**Table 1.** Basic, clinical, and circadian rhythm characteristics by CS status

Characteristics	All	Age ≥75 years		Age <75 years	
	
		CS	No CS	CS	No CS
Number of participants	1037	113	265	61	598
Basic and clinical characteristics					
Age, mean (SD), years	71.9 (7.1)	80.9 (4.1)*	78.9 (3.7)	69.4 (3.6)*	67.3 (4.3)
Male gender, *n* (%)	481 (46.4)	49 (43.4)	141 (53.2)	22 (36.1)	272 (45.5)
Body mass index (≥25 kg/m^2^), *n* (%)	263 (25.4)	17 (15.0)	59 (22.3)	18 (29.5)	169 (28.3)
Current smoker, *n* (%)	50 (4.8)	2 (1.8)	9 (3.4)	3 (4.9)	36 (6.0)
Alcohol consumption (≥30 g/day), *n* (%)	147 (14.2)	8 (7.1)	33 (12.5)	10 (16.4)	96 (16.1)
Hypertension, *n* (%)	459 (44.3)	66 (58.4)	140 (52.8)	25 (41.0)	228 (38.1)
Diabetes, *n* (%)	120 (11.7)	16 (14.3)	27 (10.4)	10 (16.4)	67 (11.3)
Sleep medication, *n* (%)	110 (10.6)	17 (15.0)	32 (12.1)	5 (8.3)	56 (9.4)
Circadian rhythm					
Bedtime, mean (SD), clock time	22:29 (1:09)	22:07 (1:13)	22:09 (1:06)	22:39 (1:05)	22:40 (1:08)
Rising time, mean (SD), clock time	6:46 (0:56)	6:52 (0:53)	6:51 (0:56)	6:35 (0:57)	6:43 (0:56)
Daytime physical activity, mean (SD), counts/min	296.4 (101.4)	265.5 (99.5)	267.3 (88.0)	337.9 (107.0)	310.8 (102.3)
Daytime light exposure, mean (SD), log lux	5.8 (1.0)	5.6 (1.1)*	5.9 (1.1)	5.8 (1.0)	5.8 (1.0)
Nighttime light exposure, median (IQR), lux	0.7 (0.1, 3.2)	0.9 (0.1, 3.2)	1.0 (0.2, 3.8)	0.4 (0.02, 2.6)	0.5 (0.1, 3.1)
UME, mean (SD), log µg	1.9 (0.7)	1.7 (0.7)	1.8 (0.7)	1.8 (0.6)	1.9 (0.7)

CS, cataract surgery; IQR, interquartile range; SD, standard deviation; UME, urinary 6-sulfatoxymelatonin excretion.

*P < 0.05 vs each reference group.

Compared to the no CS group, the CS group showed significantly higher sleep efficiency and shorter wake after sleep onset after adjustment for age (*P* = 0.024 and *P* = 0.008, respectively; Table 2). In the multivariate models, sleep efficiency was consistently significantly higher and wake after sleep onset significantly shorter in the CS group than in the no CS group, even after adjustment for age, gender, BMI, current smoking status, alcohol consumption, hypertension, diabetes, sleep medication, bedtime, rising time, daytime physical activity, daytime and nighttime light exposure, and UME (sleep efficiency: 85.8% in the CS group vs 84.4% in the no CS group, *P* = 0.042; wake after sleep onset: 45.7 min vs 50.6 min, respectively, *P* = 0.033). In addition, the associations of CS with sleep efficiency and wake after sleep onset remained significant after further adjustment for mean nighttime bedroom temperature (*P* = 0.039 and *P* = 0.032, respectively). In contrast, other actigraphic sleep parameters, such as sleep onset latency, total sleep time, and sleep-mid time, did not differ significantly between the CS and no CS groups (sleep onset latency: *P* = 0.98, total sleep time: *P* = 0.96, sleep-mid time: *P* = 0.93). In addition, UME did not differ significantly between the CS and no CS groups, even after adjustment for age, gender, bedtime, rising time, daytime physical activity, and daytime and nighttime light exposure (1.86 log µg vs 1.85 log µg, *P* = 0.90, respectively).

**Table 2.** Comparisons of objective sleep parameters by CS status using analysis of covariance

Characteristics	CS	No CS		
	
Number of participants	174	863		
	
Age-adjusted	Mean (95% confidence interval)		Difference (CS − No CS, 95% confidence interval)	*P*
Sleep efficiency, %	85.9 (84.7, 87.1)	84.4 (83.9, 84.9)	1.5 (0.2, 2.8)	0.024
Wake after sleep onset, min	44.2 (39.8, 48.6)	50.8 (49.0, 52.8)	−6.7 (−11.6, −1.8)	0.008
Sleep onset latency, log min	2.9 (2.8, 3.0)	3.0 (2.9, 3.0)	−0.1 (−0.2, 0.1)	0.49
Total sleep time, min	434.5 (423.0, 446.0)	442.9 (437.9, 447.9)	−8.4 (−21.2, 4.3)	0.19
Sleep-mid time, clock time	2:37 (2:29, 2:45)	2:38 (2:34, 2:41)	−0.3 (−9.3, 8.8)	0.96
UME, log µg	1.83 (1.73, 1.94)	1.86 (1.81, 1.94)	−0.03 (−0.1, 0.1)	0.65


Fully adjusted^a^	Mean (95% confidence interval)		Difference (CS − No CS, 95% confidence interval)	*P*

Sleep efficiency, %	85.8 (84.6, 86.9)	84.4 (83.9, 84.9)	1.3 (0.05, 2.6)	0.042
Wake after sleep onset, min	45.7 (41.7, 49.7)	50.6 (48.8, 52.3)	−4.9 (−9.3, −0.4)	0.033
Sleep onset latency, log min	2.9 (2.8, 3.1)	2.9 (2.9, 3.0)	−0.002 (−0.2, 0.2)	0.98
Total sleep time, min	441.7 (434.9, 448.4)	441.5 (438.6, 444.4)	0.2 (−7.2, 7.6)	0.96
Sleep-mid time, clock time	2:38 (2:35, 2:41)	2:37 (2:36, 2:39)	1.4 (−3.1, 3.3)	0.93
UME,^b^ log µg	1.86 (1.76, 1.97)	1.85 (1.81, 1.90)	0.01 (−0.1, 0.1)	0.90

CS, cataract surgery; UME, urinary 6-sulfatoxymelatonin excretion.

^a^Adjusted for age, gender, body mass index, smoking and drinking status, hypertension, diabetes, sleep medication, bedtime, rising time, daytime physical activity, daytime light exposure, and nighttime light exposure (per quartile increment), and UME.

^b^Adjusted for age, gender, bedtime, rising time, daytime physical activity, daytime light exposure, and nighttime light exposure (per quartile increment).

## DISCUSSION

Here, we demonstrated that CS is significantly associated with objectively measured sleep quality, as evidenced by the CS group showing better sleep quality (higher sleep efficiency and shorter wake after sleep onset) than the no CS group, after adjustment for potential confounding factors. The present study is, to the best of our knowledge, the first evidence indicating that objective sleep quality is significantly better in elderly individuals with CS than those without CS among the general population. In addition, other sleep parameters (sleep onset latency, total sleep time, and sleep-mid time) and urinary levels of melatonin metabolite did not differ significantly between the two CS groups.

The strengths of the present study included collection of extensive information on daily light exposure profiles that could be potential confounding factors, objectively measured sleep quality, and a large sample of UME measurements in the general elderly population. Intraocular lenses implanted during CS could increase capacity for light reception to the retina compared with the aged crystalline lens,^12^ although there are large individual differences in daytime light exposure levels among the elderly.^22^ Previous epidemiological studies demonstrated that elderly individuals are exposed to half as much daytime bright light (exceeding 1000 lux) as younger populations and that elderly females are exposed to half as much daytime light as age-matched males.^9^^,^^24^^,^^25^ Thus, daily light exposure profiles can be associated with aging, gender differences, and lifestyle preferences. Light reception to the retina may still be insufficient in elderly individuals with CS when the ambient light levels are too low. Thus, adjusting for daytime light exposure levels is important when considering the effect of CS on sleep quality in home settings. The present study objectively measured sleep quality, while most previous studies that identified beneficial effects of CS on sleep quality have used the subjective Pittsburgh Sleep Quality Index questionnaire.^16^^–^^18^ In this questionnaire, sleep quality is judged using seven subscales measuring different components of sleep, including sleep quality, sleep latency, sleep duration, sleep efficiency, sleep disturbance, use of sleep medication, and daytime dysfunction.^26^ However, the score of each component ranges from 0 to 3; thus, it is difficult to understand the quantitative difference in the Pittsburgh Sleep Quality Index score for each component. In addition, epidemiological data among the elderly suggests the existence of gender differences in subjective and objective sleep measures. Elderly females report shorter and poorer subjective sleep than elderly males, in contrast to actigraphic sleep measures showing poorer sleep in males.^27^ Therefore, it is preferable to analyze quantitative differences in objective sleep quality between elderly individuals with and without CS.

Detected differences in sleep quality have been assessed using a prospective cohort study, where lower actigraphic sleep efficiency was significantly associated with subsequent nursing home placement in 1664 community-dwelling elderly females.^28^ The 1.3% decrease in actigraphic sleep efficiency in the no CS group compared with the CS group in the present study is predicted to increase subsequent nursing home placement by 16.2%. However, a 1.3% difference in actigraphic sleep efficiency was detected between elderly individuals with and without CS in our study, and most individuals without CS may not have advanced cataract. Previous studies assessing the effect of CS on sleep quality were conducted using a pretest-posttest design in patients with advanced cataract.^14^^–^^18^ Taking into account these present and previous findings, the detected difference in actigraphic sleep efficiency in the present study may not be small.

In addition, our findings suggest a significant association between CS and better sleep quality even after considering increased light reception to the retina during nighttime. Daytime light exposure significantly increases nocturnal melatonin secretion and improves sleep quality,^9^ although in the present cross-sectional study we did not detect a significant difference in UME or some of the actigraphic sleep parameters, such as sleep onset latency, total sleep time, and sleep-mid time, between elderly individuals with and without CS. This may be explained by previous evidence that nighttime light exposure significantly suppresses melatonin secretion and impaired sleep quality.^29^^,^^30^ The intraocular lens implanted by CS may increase the capacity for light reception to the retina not only during daytime but also during nighttime compared with an aged crystalline lens; therefore, nighttime light exposure to the retina in elderly individuals with CS may be higher than those without CS. However, the present final multivariate statistic models regarding the associations of CS with actigraphic sleep quality included nighttime light exposure as a confounding factor, suggesting that the association between CS and better sleep quality is independent of nighttime light exposure, although a residual effect of increased nighttime light exposure on melatonin levels and sleep quality in elderly individuals with CS may exist.

The present study included some limitations. First, CS history was ascertained using a self-reported questionnaire rather than objective measurement, possibly leading to the misclassification of CS status of some participants. However, sufficiently high agreement observed between self-reported questionnaire and objective measurement of CS history among the initial 194 participants suggested that misclassification was rare. Second, we have no information about the intraocular lens implanted at CS (ie, clear or yellow intraocular lens). The intraocular lenses of some individuals with CS may be yellow, which filter short wavelengths (below 500 nm) that are most sensitive to intrinsically photosensitive retinal ganglion cells.^12^ This means that the present 1.3% difference in actigraphic sleep efficiency may underestimate the effect if all individuals had received clear intraocular lenses. Also, we have no information related to severity of cataract before surgery, visual acuity before and after CS, CS done for one eye or both eyes, and the period since CS. A previous study indicated that sleep quality as well as visual acuity was improved even at 1 month after CS,^15^ whereas some residual confounding effect of these may exist. In addition, the statistical models did not include information on sleepiness-related medications and sleep-related conditions, such as antiallergic drugs, chronic pain, and restless leg syndrome. Finally, the present study was conducted with a cross-sectional design; therefore, some issues, such as causality, could not be assessed. To better understand the effect of CS on melatonin secretion and objective sleep quality, a well-designed randomized controlled trial is needed.

In conclusion, the present study demonstrated that CS is significantly associated with objectively measured sleep quality among a community-dwelling elderly population, but that urinary levels of melatonin metabolite do not differ between elderly individuals with and without CS. These associations are independent of potential confounders, such as age, gender, physical activity, and daily light exposure profiles.

## ONLINE ONLY MATERIAL

Abstract in Japanese.
