# Supplementary material for: Comparisons of Objective Sleep Quality Between Elderly Individuals With and Without Cataract Surgery: A Cross-Sectional Study of the HEIJO-KYO Cohort
Source: J Epidemiol. 2015 Aug 5;25(8):529–35. doi: 10.2188/jea.JE20140201 (PMC4517991; doi:10.2188/jea.JE20140201)
Supplement: Abstract in Japanese. [file je-25-529-s001.pdf]

高齢者における白内障手術の有無による客観的な睡眠の質の比較： 平城京コホートスタディの横断解析

大林賢史[1], 佐伯圭吾[1], 宮田季美恵[2], 西智[2], 刀根庸浩[3], 緒方奈保子[2], 車谷典男[1]

[1]奈良県立医科大学 地域健康医学講座, [2]奈良県立医科大学 眼科学講座, [3]奈良県立医科大学 産学官連携推進センター

背景
白内障手術により網膜への光受容は劇的に増加する。先行研究で白内障手術が主観的に測定された睡眠の質を改善することが報告されているが、白内障手術と客観的睡眠の質の関連は十分に分かってない。

方法
白内障手術と客観的に測定された睡眠の質の関連を明らかにするために、我々は高齢者 1037 人（平均年齢 71.9 歳）を対象に横断研究を実施した。白内障手術の有無に加えて、アクチグラフによる睡眠の質、夜間尿中メラトニン主要代謝産物排泄量（6-sufatoxymelatonin）、および環境照度を測定した。

結果
白内障手術群（n = 174）は非白内障手術群（n = 863）と比較して、年齢、性別、BMI、喫煙、飲酒、高血圧、糖尿病、睡眠薬の使用、入床時刻、離床時刻、日中身体活動量、日中および夜間の光曝露量を調整した後でも、有意に睡眠効率が高く、睡眠後覚醒時間が短かった（睡眠効率：85.8% vs. 84.4%, P = 0.042; 睡眠後覚醒時間：45.7 分 vs. 50.6 分, P = 0.033）。対照的に、夜間尿中メラトニン主要代謝産物排泄量、睡眠潜時、総睡眠時間、および睡眠中央時刻は 2 群で有意な差を認めなかった。

結論
一般高齢者集団において、白内障手術の有無は睡眠効率や睡眠後覚醒時間と有意に関連していた。しかし、夜間尿中メラトニン主要代謝産物排泄量は白内障手術群と非白内障手術群で有意な差を認めなかった。また、これらの関連は日常の光曝露量と独立していた。

キーワード
睡眠の質、白内障手術、サーカディアンリズム、アクチグラフ、メラトニン
